# Physical therapy for facial nerve paralysis (Bell's palsy): An updated and extended systematic review of the evidence for facial exercise therapy

**DOI:** 10.1177/02692155221110727

**Published:** 2022-07-05

**Authors:** Amir J Khan, Ala Szczepura, Shea Palmer, Chris Bark, Catriona Neville, David Thomson, Helen Martin, Charles Nduka

**Affiliations:** 1 Department of Economics, 119703 Institute of Business Administration, Karachi, Pakistan; 2 Centre for Healthcare Research, 2706 Coventry University, Coventry, UK; 3 Centre for Care Excellence, 2708 Coventry University & University Hospital Coventry & Warwickshire, Coventry, UK; 4 Lanchester Library, 2706 Coventry University, Coventry, UK; 5 8962 Queen Victoria Hospital NHS Foundation Trust, East Grinstead, West Sussex, UK; 6 6718 St Helens and Knowsley Teaching Hospitals NHS Trust, Liverpool, UK

**Keywords:** systematic review, Bell's palsy, facial exercise therapy, telerehabilitation, COVID-19

## Abstract

**Objective:**

To conduct a systematic review of the effectiveness of facial exercise therapy for facial palsy patients, updating an earlier broader Cochrane review; and to provide evidence to inform the development of telerehabilitation for these patients.

**Data Sources:**

MEDLINE, EMBASE, CINAHL, Cochrane Library, PEDro and AMED for relevant studies published between 01 January 2011 and 30 September 2020.

**Methods:**

Predetermined inclusion/exclusion criteria were utilised to shortlist abstracts. Two reviewers independently appraised articles, systematically extracted data and assessed the quality of individual studies and reviews (using GRADE and AMSTAR-2, respectively). Thematic analysis used for evidence synthesis; no quantitative meta-analysis conducted. The review was registered with PROSPERO (CRD42017073067).

**Results:**

Seven new randomised controlled trials, nine observational studies, and three quasi-experimental or pilot studies were identified (*n*  =  854 participants). 75% utilised validated measures to record changes in facial function and/or patient-rated outcomes. High-quality trials (4/7) all reported positive impacts; as did observational studies rated as high/moderate quality (3/9). The benefit of therapy at different time points post-onset and for cases of varying clinical severity is discussed. Differences in study design prevented data pooling to strengthen estimates of therapy effects. Six new review articles identified were all rated critically low quality.

**Conclusion:**

The findings of this targeted review reinforce those of the earlier more general Cochrane review. New research studies strengthen previous conclusions about the benefits of facial exercise therapy early in recovery and add to evidence of the value in chronic cases. Further standardisation of study design/outcome measures and evaluation of cost-effectiveness are recommended.

## Introduction

Bell's palsy is the most common form of acute spontaneous peripheral facial paralysis, with poor recovery affecting a patient's long-term quality of life.^1^ The cause still unclear,^2^ although the condition has long been associated with reactivation of latent virus infection,^3^ with evidence showing a rise in incidence in the United States thought to be linked to increasing herpes infection rates.^4^ The majority (60%) of these facial nerve paralysis cases are Bell's palsy,^5^ and this condition affects 11–40 people per 100,000 in the population each year, most commonly in the age group 30–45.^6^ It is also estimated that one in sixty individuals will be affected over the course of their lifetime.^7,8^ Population studies show that facial palsy is more commonly associated with people who are immunocompromised or pregnant, or those with obesity, hypertension, diabetes, or upper respiratory conditions.^1,7,9^ With the appearance of COVID-19 caused by SARS-CoV-2, reports are also emerging of Bell's palsy as a presenting symptom this for infection.^10,11^ Although most patients eventually recover without any treatment,^6,12^ one in three (29%) have a poor recovery resulting in a permanent deficit of facial function.^6,13^ This can affect important functions such as eating and speaking,^14^ or non-verbal communication mediated by expression of emotion (e.g. smiling),^15^ as well as chronic facial pain.^6,16^ Patients living with incomplete recovery experience long-term psychological distress and depression, with many moving away from public-facing roles resulting in social alienation.^6,16–19^

The evidence base for therapy in acute and chronic cases remains limited. Currently, a range of treatment options may be offered, with a recent study identifying considerable variation in the care pathways experienced by patients in the United Kingdom.^20^ These range from medication and physical therapies to surgery. Evidence of the effectiveness of surgical interventions remains limited, with a Cochrane systematic review reporting insufficient proof of benefit and further trials judged to be unlikely.^21^ Cochrane reviews have identified one pharmaceutical treatment (prednisolone) as an effective treatment (versus placebo) for Bell's palsy, but only if administered within 72 h of symptom onset.^22–24^ Use of adjunctive antiviral therapy was reported to be of uncertain value,^22^ although more recent evidence of its value in the treatment of Ramsay Hunt syndrome, the second most common cause of facial palsy, has emerged.^25^ In addition to medication, a number of physical therapy options are available. Facial exercise therapy (facial neuromuscular retraining) is the most widely evaluated.^26–31^ A Cochrane review of physical therapies published in 2011 reported some evidence that facial exercise therapy could improve facial function for moderate paralysis and chronic cases and reduce sequelae in acute cases, but it recommended the need for further studies.^32^ We have therefore undertaken a systematic review to identify and appraise any further studies that have examined the effectiveness of facial exercise therapy. Updated evidence on effectiveness is required to inform the planned development of facial remote activity monitoring eyewear to enable telerehabilitation in a patient's own home.^20,33^

## Aims and methods

The review aimed to identify and assess recent evidence of the effectiveness of facial exercise therapy, draw out any clinically relevant findings, and identify future research needs. The review protocol was submitted for peer-review to the International Prospective Register of Systematic Reviews (PROSPERO), ID: CRD42017073067.^34^ A mixed-methods approach to evidence synthesis was specified in the protocol to allow for the inclusion of different study designs, in addition to randomised controlled trials.^35,36^ The peer-reviewed protocol also specified the inclusion of evidence from post-2011 reviews to maximise the evidence identified. There was strict adherence to international guidelines on Preferred Reporting Items for Systematic Reviews and Meta-Analyses (PRISMA).^37^

Literature searches were undertaken by an Academic Librarian (CB) qualified in systematic reviews. Databases searched included MEDLINE, EMBASE, CINAHL, Cochrane Library (Database of Systematic Reviews and Central Register of Controlled Trials) and PEDro; plus the Allied and Complementary Medicine Database). The latter was not included in the original Cochrane review. Searches were replicated covering the period 1 January 2011 to 30 September 2020, allowing for a slight overlap with the search end date (28 February 2011) of the earlier Cochrane review.^32^ Tailored search strategies were used for the six databases and details are presented in Appendices 1–6.

Abstracts were screened against predetermined inclusion/exclusion criteria (see Table 1), and abstracts shortlisted against these. Study designs included both individual studies and reviews. All identified records were first imported into the Refworks Pro database. A separate file was prepared for each database. Full articles were downloaded and shortlisted based on content by two independent reviewers (blind review). Shortlists were compared by a third reviewer and any differences resolved by discussion between the two original reviewers; if arbitration was required this was provided by another member of the review team (AK, AS, CT, HM, and DT participated in this process).

**Table 1.** Selection criteria.

	Inclusion Criteria	Exclusion Criteria
Population	Includes adults with a diagnosis of Bell's palsy (idiopathic facial palsy)/facial palsy.	Patients aged <18 yrs. Bell's palsy patients not included
Study design	Randomised controlled trial, Quasi-experimental studies, Pilot or feasibility studies, Non-experimental observational (cross-sectional, case-series), Reviews.	Individual case study,
Intervention	All types of facial exercise interventions for facial palsy i.e. such as strengthening and stretching; endurance; therapeutic and facial mimic exercises (“mime therapy”). Facial exercise therapy alone, with biofeedback, or combined with another treatment.	Interventions do not include facial exercise therapy.
Comparator	No treatment, placebo treatment, drug treatment, or other physical therapy interventions.	No comparator
Outcomes	House–Brackmann/Sunny Brook Facial Grading Systems; time to recovery; residual symptoms (motor synkinesis, contracture, hyperkinesia, facial spasm or crocodile tears); incomplete recovery after one year; quality of life and disability; adverse events attributable to therapy e.g. pain or worsening of condition; factors limiting efficacy of facial exercises	Does not report an evaluation or include descriptions of health outcome measures
Other	Abstract & Article in English language	Article not in English language
	Papers published in peer-reviewed journals, irrespective of country	Publications in non-peer-reviewed journals

Shortlisted articles were read carefully and assessed by two independent readers (AK and DT) for inclusion in the review. Some articles were discarded if these repeated information provided in another version. Details of all articles were entered into an Endnote library and bibliographies were scrutinised to identify possible additional studies. Screening and shortlisting data were presented as a PRISMA flow diagram.^37^

For data extraction and management, articles were organised into two groups (i) individual primary research studies and (ii) reviews. Summary Tables were piloted for data extraction from both types of articles. Common information extracted from all articles included author names, publication date, and country of publication. For individual research studies, additional data recorded included: study design; sample size & patient demographics; details of clinical diagnoses/conditions; details of intervention and comparator(s); outcome measures; main findings; and conclusions. For review articles, data extracted also included type and number of studies reviewed, publication dates, and total number of patients. Information was extracted and entered into the Summary Table initially by two author (AK, DT). This was then checked and amended and expanded where necessary by other reviewers, separately for individual studies (CT, HM, AS) and for reviews (CN, SP).

Quality assessment was undertaken using validated tools. For individual research studies, quality and risk of bias were assessed using GRADE (Grades of Recommendation, Assessment, Development, and Evaluation) appraisal tools.^38,39^ Assessment was performed independently by pairs of reviewers (AK, CT, HM, and DT). Studies were categorized into three levels of bias (low, unclear, and high risk of bias); a third researcher (AS) assisted with any disagreements that arose. For the published reviews shortlisted, quality was assessed using AMSTAR-2 by two independent reviewers (SP, CN). Confidence in the quality of each review was categorised as high, moderate, low, or critically low, based on the number of critical and non-critical flaws identified.^40^

In preparation for final analysis, all papers were read in full, with data and results extracted, regardless of the study design (AK, CT, HM, and AS participated in this process). For individual studies, data entered into the Summary Table included study design, diagnoses, therapies considered, results, limitations, and study quality rating. A further table collated the main findings and conclusions drawn (CT, HM, AS, SP, CN). As anticipated, authors used differing study designs, papers considered a mix of therapies and controls, and adopted different outcome measurement tools. Statistical combination of numerical data from separate studies to strengthen review conclusions (meta-analysis) was not possible due to variations in study design and quality. A thematic analysis framework was therefore used to create a descriptive synthesis and address the heterogeneity of studies identified.^41^ Where available, the results of individual randomised controlled trials were reported separately, as were the findings of cohort studies. The authors coded all the studies before discussing and agreeing on descriptive categories and themes for synthesis.

## Results

**Figure 1.**
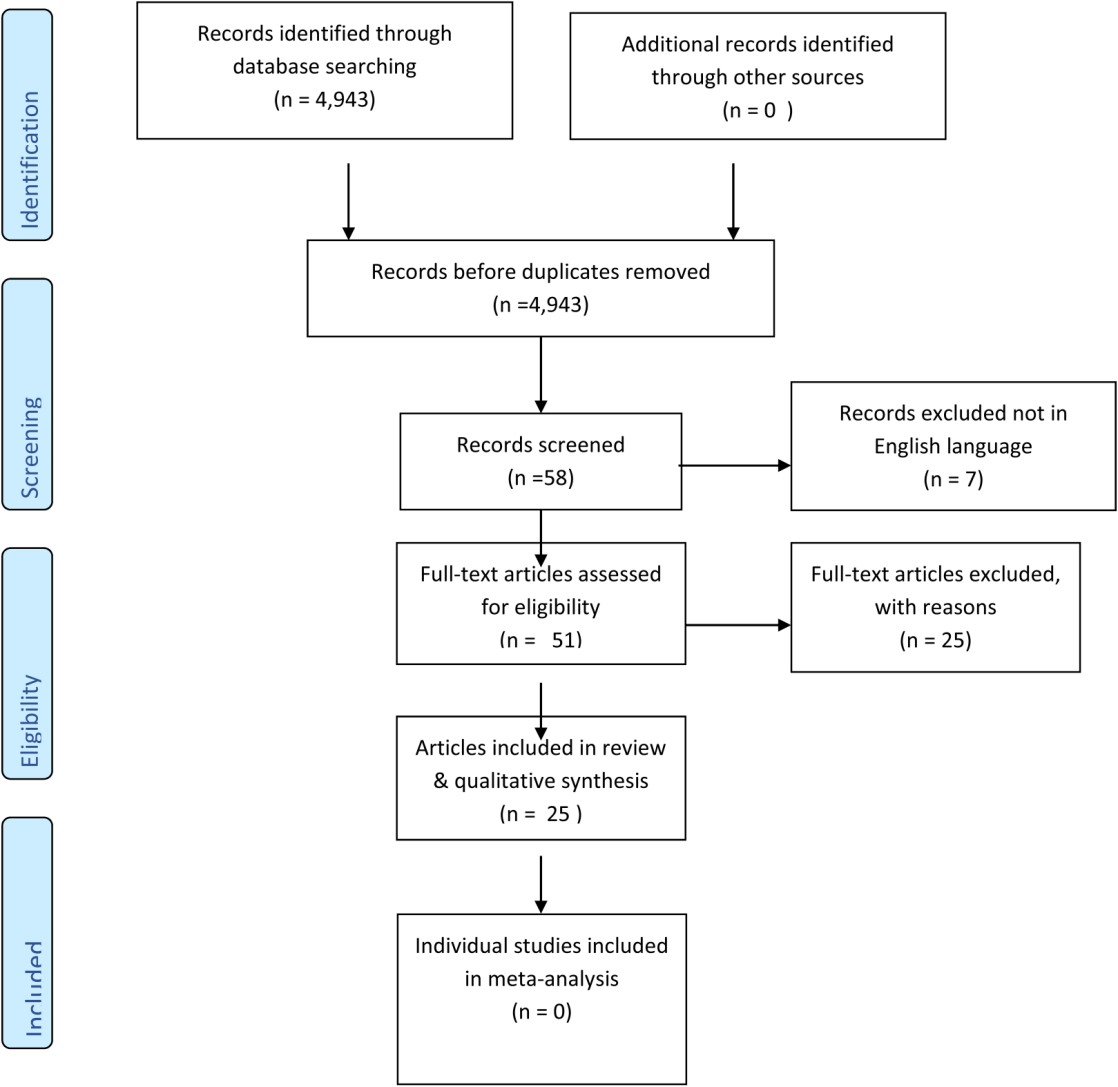
Preferred Reporting Items for Systematic Reviews and Meta-Analyses (PRISMA) flow diagram outlining the study design.

Figure 1 presents a PRISMA flow diagram summarising the review process. The database searches identified a total of 4943 titles and abstracts for possible inclusion. A total of 58 articles were shortlisted for the review; seven further articles were discarded because only the abstract was in the English language. In total, 51 articles were selected for reading the full text; 22 further papers were removed, with reasons recorded, and three further items were discarded because they were narrative revisions over time of the same study, making a total of 25 publications excluded. This left 25 articles for inclusion in the final review; 19 were individual studies; and 6 reviews (in addition to the original Cochrane review).

Table 2 (individual research studies) and Table 4 (reviews) present summaries of the characteristics of these 25 new studies identified for the review. Individual studies originated from 10 different countries, including Canada, Iran, Italy, Japan, Portugal, Saudi Arabia, South Africa, South Korea, Turkey, and the United States, emphasising the world-wide interest in physical therapy for facial palsy patients. The six new review articles originated from Australia,^42^ Brazil,^43^ Iran,^44^ Italy,^45^ New Zealand,^46^ and Portugal.^47^

**Table 2.** Summary table: Characteristics of included individual research studies.

Authors (date/country)	Study design	Participant characteristics	Included diagnoses/conditions	Therapeutic interventions	Outcome measurement tools	Study Quality (GRADE)
Alakram & Puckree^48^ (2011; South Africa)	A two-group pre-test post-test experimental design.	16 patients with Bell's palsy referred to 3 hospitals	Bell's Palsy of less than 30 days duration.	Both groups treated with heat, massage, facial exercises and given a home programme. Intervention group had added electrical stimulation.	Facial Disability Index (FDI)	Low
Alayat et al.^60^ (2014; Saudi Arabia)	Randomised controlled trial double blind, pre- post comparison.	48 patients with Bell's palsy mean age 43 ± 9.8 years. Randomly assigned into three groups: high intensity laser therapy (HILT), low level laser therapy (LLLT), and exercise group	Any patient who had unilateral Bell's palsy either on the right or left side. sub-acute stage of illness 3–5 days after the acute onset subsided	Intervention 3 times per week for 6 weeks. HILT and LLLT group received facial massage and exercises after laser therapy. All 3 groups continued exercises at home.	Facial disability index (FDI) and House–Brackmann Scale (HBS) measured before as well as 3 and 6 weeks after treatment.	High
Azuma et al^59^ (2012; Japan)	Prospective clinical study	13 patients; 8 patients with Bell's palsy and 5 with herpes virus infection	Patients with Bell's palsy and with herpes zoster showing facial synkinesis	Single dose of botulinum-A toxin followed by facial rehabilitation. Then daily facial biofeedback rehabilitation with a mirror at home.	Percent of eye opening during 3 designated mouth movements (lip pursing, teeth baring, and cheek puffing):/	Low
Barth et al^54^ (2020; USA)	Chart review of patients’ charts, before and after treatment	45 patient selected at random from pool of approximately 100 patients treated for facial palsy between 1997 and 2008	25 patients with idiopathic facial palsy.	10 patients received facial physical rehabilitation. 15 patients received mirror book therapy in conjunction with standard facial rehabilitation.	Sunnybrook Facial Grading System (SB-FGS) Observational Scale and Facial Disability Index (FDI) measured pre-treatment and post-treatment.	Medium
Dalla Toffola et al^51^ (2012; Italy)	Cohort study on retrospective clinical records.	102 patients (63 males, 61.8% and 39 females, 38.2%), mean age 48 years (range 12–80) consecutively assessed between January 2003 and December 2010.	Patients with Bell's palsy (54 right and 48 left) clinically evaluated within one month of onset.	Rehabilitation treatment personalised based on EMG/ENG performed 3–4 weeks after onset. 29 patients with neurapraxia not given treatment. 38 patients electromyographic biofeedback (EMG-BFB) 35 treated with mirror visual feedback (MIRROR-BFB)	Estimates of clinical improvement at 12 months (House scale), recovery time, development of synkinesis, number of treatment sessions	High
Dalla Toffola et al^52^ (2014; Italy)	Cohort study of 30 patients followed up for three years	47 postsurgical patients	Patients with complete lateral facial palsy (House-Brackmann grade VI)	Home rehabilitation programme involving mirror visual feedback following XII-VII anastomosis	House-Brackmann (HBS) grade at 12, 18 and 36 months after surgery	Low
Di Stadio et al^66^ (2020; Italy)	Randomised case-control study	20 patients over 18 years with Bell's palsy	Patients with unilateral Bell's palsy within 5 days of symptoms onset. MRI or clinical evidence of Ramsay Hunt syndrome	Group A underwent exclusively Kabat rehabilitation, and group B patients treated by combining facial taping and Kabat.	Arianna Disease Scale (ADS) at baseline, 1 week, 1 month and 3 months after treatment.	Low
Ferreira et al^61^ (2016;Portugal)	A prospective single-blinded randomised controlled trial.	73 patients, aged ≥18 years: *N* = 31 facial neuromuscular training (FNT); median age 49.0 years; *N* = 42 corticosteroids + FNT (median age 37.5 years).	Patients with unilateral Bell's palsy.	Corticosteroids plus facial neuromuscular training (FNT) vs FNT only. Oral corticosteroids treatment within 72 h Bell's palsy onset, and FNT within 10 days after onset	House–Brackmann Scale (HBS) and Sunnybrook Facial Grading System (SB-FGS) before and 6 weeks after treatment.	High
Fujiwara et al^53^; (2018; Japan).	Observational cohort study	37 hospital patients with peripheral facial palsy: 15 male, 22 female; median age 57 (range 28–79)	15 patients with Bell's palsy, 12 with Ramsay Hunt syndrome. All patients showing synkinesis at 6 months after onset	All patients had physical therapy involving 30 min home training daily, and mirror biofeedback.	Sunnybrook Facial Grading System (SB-FGS), asymmetry in eye opening width, synkinesis at 6, 9 and 12 months.	Medium
Karp et al^55^ (2019; USA)	Retrospective case review of hospital referrals	76 Bell's palsy patients aged 20 to 89 years (mean = 49.5 years) referred between 1995 and 2016.	Patients diagnosed with facial nerve paralysis ≥ 12 months (range 12–384 months) prior to initiation of physical therapy	All patient had at least 2 sessions of directed physical therapy by a single therapist These includes: neuromuscular retraining, stretching/massage, and active exercise.	Improvement in Facial Grading System (FGS) scale after physical therapy	Low
Kim et al^56^ (2011; South Korea)	Group comparison on selected participants.	26 patients receiving Western-oriented therapy (12 male; 14 female). Mean age 47.8 ± 14.4 years.	19 patients with facial palsy on right side; 7 on the left.	Symmetric self-performed facial muscle exercises (SSFE) in addition versus standard Western therapy alone; 12 SSFE vs 14 controls. SSFE patients exercised 3 times per day for 4 weeks.	House–Brackmann Scale (HBS) and Yanagihara's Unweighted Grading System before therapy and 4 weeks after completion.	Medium
Martineau et al^49^ (2020; Canada)	Pilot study, small sample comparison group.	10 patients	Patients with acute moderate-to-severe, severe, and total Bell's palsy.	Mirror Effect PLUS Protocol (MEPP) specifically designed for acute Bell's palsy	Sunnybrook Facial Grading System (SB-FGS), and Facial Disability Index (FDI)	Low
Monini et al^64^ (2011; Italy)	Randomised controlled trial	20 consecutive patients recovering from facial palsy (12 females, 8 males, age range 18–67 years)	Patients recovered from acute-onset facial palsy with a residual HB II and III grade observed over 2-year timeframe. Kabat physical rehabilitation for at least 1 year.	NeuroMuscular Retraining Therapy (NMRT) alone versus additional pre-treatment with Botulinum toxin type A (BTX-A).	House–Brackmann Scale (HBS) supported by video FaceO-Gram system; Sunnybrook Facial Grading System (SB-FGS) day 7 and day 90 post BTX-A treatment.	Medium
Monini et al^58^ (2017; Italy)	Observational study, patients observed in hospital ED (and assigned to one group) between January 2005 and June 2014.	104 patients presenting at tertiary hospital between January 2005 and June 2014	Patients presenting with House-Brackmann Grade IV or V.	Medical treatment only (corticosteroids for 10 consecutive days, subsequently tapered off) vs Group receiving Medical treatment + Kabat physical rehabilitation	House–Brackmann (HBS) grade shift value; speed of recovery (days to stable or final clinical recovery; and reduced version of FaCE Scale questionnaire (Beta FaCE Scale)	Medium
Nicastri et al^62^ (2013; Italy)	Randomised controlled trial	87 patients aged 15–70 years presenting from June 2008 to May 2010.	Unilateral Bell's Palsy severity House-Brackmann (HB) scale grade IV to VI within 10 days of onset; onset of steroid treatment within 48 h after initial symptoms.	Physical therapy in association with standard steroid treatment versus pharmacological therapy only.	Proportion patients HBS grade ≤ II at end of 6-month. Secondary outcomes: time to reach HBS grade ≤ II; differences in mean Sunnybrook (SB-FGS) total score; proportion patients synkinesis subscore zero at 6-months.	High
Ordahan & Karahan^65^ (2017; Turkey).	Randomised controlled study	46 patients (mean age 41 ± 9.7 years)	Patients admitted to hospital in Turkey with unilateral Bell's Palsy.	Low-level laser therapy in conjunction with conventional facial exercise vs facial exercise only.	Facial Disability Index (FDI) measured pre-treatment and 3-week and 6-week post-treatment	Medium
Pourmomeny et al^67^ (2015; Iran)	Randomised controlled trial	34 patients referred to 3 university hospitals	Facial synkinesis	Both groups EMG biofeedback for 4 months. One group single dose of botulinum toxin type A (BTXA); control group received saline	Facial Grading System (FGS) using Photoshop software and videotape.	Low
Rodriguez et al^50^ (2012; Canada)	Pilot study: prospective, randomised controlled trial.	27 patients presenting at two Emergency Departments, 14 male; 13 female (mean age 48 ± 15.7 years)	Patients with unilateral, idiopathic cranial nerve VII paresis	Patients randomized to fine-motor eye exercises (*n* = 18) or to do no exercise (*n* = 9) for a period of four weeks.	Orbicularis oculi muscle strength measured in both eyes at baseline, two weeks and four weeks. Functional recovery gauged by total muscle function determined to be clinically significant.	Low
Tuncay et al^63^ (2015; Turkey)	Randomised controlled trial	60 patients presenting at hospital between March 2010 and May 2012, 29 male; 31 female (mean age 44.8 ± 17.6 years, range 18–79 years)	Patients with Bell's Palsy only; new onset of idiopathic facial paralysis within 48 h.	Physical therapy (facial expression exercises via a mirror), 5 times per week over 3 wks. Group 2, electrical stimulation treatment in addition to the physical therapy.	House-Brackmann (HBS) scale and Facial Disability Index (FDI); electrophysiologic outcome compound muscle action potentials (CMAPs) at 4 and 12 wks.	High

**Table 4.** Summary table: Characteristics of included systematic reviews.

Authors (date/country)	Review Type (*search dates*)	Articles Reviewed (*publication dates*)	Patient Characteristics *(Total Number)*	Therapeutic interventions	Outcome measurement tools	Review Quality (AMSTAR-2)
Teixeira et al^32^ (2011; Brazil)	Review of randomized and quasi-randomized controlled trials *(database inception - 2011)*	12 randomised or quasi-randomised controlled trials included in review, *(published 1958–2010)*	Patients of any age with a diagnosis of Bell's palsy and all degrees of severity *(Total 872 patients)*	Exercise (3 trials; 199 patients) Electrical stimulation (4 trials; 313 pts) Compare/combine physical therapy with acupuncture (5 trials; 360 pts).	Incomplete recovery 6 & 12 months; Motor synkinesis at 6 months; Crocodile tears, facial spasm at 6 months; Adverse effects of intervention	High
Pereira et al.^43^ (2011; Brazil).	Review of RCTs (facial exercises with/without mirror biofeedback) *(database inception - 2010)*	6 RCT studies included in review (1 suitable for meta-analysis) *(published 1991–2009)*	Patients with all forms of facial palsy (67% Bells’ palsy, 13% Herpes Zoster) *(Total 196 patients)*	Mirror biofeedback; Conventional facial exercise therapy	HBS; FDI; SB-FGS; linear measurement of facial movement; CMAP amplitude; video analysis; synkinesis	Critically low
Baricich et al.^45^ (2012; Italy) Have full article now	Review of randomized or quasi randomized controlled trials, case control, cohort studies and case series > 6 patients *(searched 1990–2010)*	15 articles included in review: 7 RCTs, 4 pilot studies, 1 preliminary study, 3 retrospective studies *(published 1991–2010)*	Patients with all types of peripheral facial nerve palsy *(Total 214 patients)*	Electrostimulation; Mime therapy; Biofeedback; Proprioceptive Neuromuscular Facilitation (PNF); Neuromuscular re-education	9 studies used HBS; 3 studies used SB-FGS; 1 study used FDI; others included EMG, video analysis, CMAPs.	Critically low
Holland et al.^46^ (2014; New Zealand)	Review of RCTs and systematic reviews *(database inception - Oct 2013)*	1 RCT and 2 systematic reviews included in review *(RCT published 2006; Reviews published 2007 & 2008)*	Patients with a diagnosis of peripheral facial paralysis or Bell's palsy *(Total 48 RCT patients; 679 Review patients)*	Antiviral drugs, corticosteroid drugs; Hyperbaric Oxygen Therapy; Facial Re-training (Mime Therapy and Exercise)	FDI; Motor synkinesis; Time to begin/complete recovery	Critically low
Pourmomeny & Asadi^44^ (2014; Iran).	Review of RCTs *(searched 1980 - mid-2013)*	6 RCTs included in review *(published 2003–2013)*	Patients with Bells’ palsy/facial nerve palsy (excluded immediate treatment that precluded spontaneous recovery) *(Total 269 patients)*	Mirror biofeedback; Mime therapy; EMG biofeedback; Physiotherapy	HBS; SB-FGS; linear measurement of facial movement; video analysis	Critically low
Ferreira et al^47^ (2015; Portugal)	Review of randomized and quasi-randomized controlled trials *(database inception - Aug 2013)*	4 studies included in review *(published 2009–2013)* but designs not clearly reported.	Patients older than 15 years with a clinical diagnosis of Bell's palsy *(Total 143 patients)*	Compared facial physical therapy combined with standard drug treatment against a control group with standard drug therapy alone.	Primary outcome: HBS. Secondary outcomes: adverse events; CMAPs residual symptoms; SB-FGS	Critically low
Fargher & Coulson^42^ (2017; Australia)	Review of randomized and quasi-randomized controlled trials *(database inception - Aug 2016)*	5 RCTs included in review (4 acute phase & 1 chronic) *(published 1958–2016)*	Patients with Bell's palsy (4 trials during acute recovery; 1 trial chronic patients) *(Total 258 patients)*	Electrical stimulation therapy for patients with acute or chronic facial nerve palsy	HBS; SB-FGS; FDI; Facial Paralysis Recovery Profile; Facial Paralysis Recovery Index; Video-based motion analysis	Critically low

CMAPs: Compound Muscle Action Potentials; EMG: electromyography; FDI: Facial Disability Index; HBS: House–Brackmann Scale; SB-FGS: Sunnybrook Facial Grading System.

*Characteristics of included studies:* The 19 individual studies shown in Table 2 included, seven randomised controlled trials (total no. of subjects  =  354); one quasi-experimental study (*n* = 16 patients)^48^; two pilot studies (*n* = 37 patients)^49,50^;and nine non-experimental observational studies (*n* = 447 patients). Observational studies varied in their design: three were cohort studies (*n* = 169 patients)^51–53^; two were retrospective studies (*n* = 101 patients)^54,55^; three used group comparisons (*n* = 164 patients)^56–58^; and one a small clinical study of 13 patients.^59^ In total, the research studies identified reported the impact of physical therapy in a total 854 participants (age range 12–80 years old).

*Methodological quality and risk of bias*: Consensus ratings of the methodological quality of studies (GRADE) are shown in the final column of Table 2. Five of the 19 individual studies were rated as high quality; four of these were randomised controlled trials^60–63^; and one was a cohort study.^51^ A further six studies were judged to be of moderate quality. Two were randomised controlled trials,^64,65^ and four were non-experimental observational studies.^53,54,56,58^ The remaining nine studies were rated as low methodological quality. Two were randomised trials,^57,66^ one was a quasi-experimental study,^48^ and two were small pilot studies.^49,50^ In terms of methodological flaws all, except one study,^60^ were at risk of bias for failing to blind participants when outcomes were based on patient-reported measures. One high-quality study failed to achieve the required sample size, with a significant number of severe facial palsy patients in the control group dropping out in order to access physical therapy which was shown to be improving outcomes.^62^

*Patient characteristics (diagnoses/conditions):* There was a high level of heterogeneity in the characteristics of patients included in the studies as shown in Table 2. Where specified, there was considerable variation in time post-onset of facial palsy at study entry. Six studies focused on patients in the acute phase, ranging from 48 h,^63^ five days,^66^ ten days,^62^ to one month since onset.^48,51^ One study recruited patients in the ‘sub-acute phase’, that is, 3–5 days after the acute onset subsided.^60^ A further two studies focused on chronic stages of recovery, that is, 2–30 years since onset.^55,64^ The remaining 11 studies did not pre-specify time post-onset at study entry. Individual studies also varied in the types of facial palsy patient recruited. All studies included Bell's palsy patients. Three studies also recruited some Ramsay Hunt syndrome patients.^45,53,66^ Where specified, all patients had unilateral facial palsy, although some studies did not state the type. Some studies recorded patients’ level of facial synkinesis at entry.^53,57,59^ Others focused on the severity of facial palsy; this ranged from Grade II to VII.^50,52,58,62,64^

*Therapies Evaluated:* Only two studies evaluated the use of facial muscle strengthening exercises on their own.^50,55^ A further five studies evaluated facial exercise therapy combined with biofeedback (via mirror or other device)^49,51–54^; and one evaluated exercise with added facial taping.^66^ One paper compared Western facial exercise therapy with or without the addition of a Korean variant of facial muscle exercises.^56^ The remaining studies reported the effects of combining facial exercise therapy with another form of treatment. Two studies explored the use of laser treatment before or in conjunction with facial exercises,^60,65^ and two research teams assessed the addition of electrical stimulation to physical therapy.^48,63^ Three papers examined physical therapy with and without corticosteroids^58,61,62^; and three studies considered Botox added to facial therapy.^57,59,64^

*Outcome measures reported:* Individual studies used three main validated measurement tools (see Table 2). Further details about these and patterns of combined use are presented in Appendix 7. The House–Brackmann Scale (HBS) was the most common method used to record improvements in functional recovery following therapy (*n*  =  9 studies). A further six studies incorporated the Sunnybrook Facial Grading Scale (SB-FGS) to measure the severity of facial paralysis symptoms. A patient-rated outcome measure of the disability resulting from facial palsy, the Facial Disability Index (FDI), was used in six studies; one study reported separate results for the physical elements (FDIP) and social elements (FDIS) of the FDI.^54^ The five remaining studies utilised a different single measure; two used an alternative validated facial grading system,^55,67^ and three used another unvalidated measure.^50,59,66^ All these five studies were assessed to be of low quality. Outcome measures used and their timing varied in the four high-quality randomised controlled trials,^60–63^ meaning comparable data were not available for meta-analysis to be undertaken.^68^ The choice of outcome measure(s) was not indicative of the final quality (GRADE rating) of studies, although highly rated studies tended to use a combination of measures. For the randomised controlled trials, domains in which GRADE identified critical flaws are identified in Table 3.

**Table 3.** Results reported in individual research studies.

Authors (date/country)	Main Findings – Recorded Outcomes and/or Functional improvements	Study Conclusions
Alakram & Puckree^48^ (2011; South Africa)	Effects of electrical stimulation, quantified by FDI, clinically significant but not statistically significant. The experimental group also received electrical stimulation. The FDI of the control group improved between 17.8% and 95.4% with a mean of 52.8%. The improvement in the experimental group ranged between 14.8% and 126% with a mean of 49.8%.	Limited, not significant improvement during acute phase of Bell's palsy through addition of electric stimulation to heat, massage, exercises and home programme.
Alayat et al^60^ (2014; Saudi Arabia)	Analysis of the HBS and FDI scores with Mann–Whitney U-test after 6 weeks of treatment showed a significant difference between the treatment groups. Greatest effect observed in high intensity laser therapy group, followed by low level laser therapy group, and exercise group. HILT/HBS:25.97–36.44 Exercise/HBS:23.13–14.13 HILT/PFDI:20.06–39.74 Exercise/PFDI:26.84–10.84	Further improvement in outcomes of exercise therapy by addition of high intensity laser therapy or low level laser therapy. GRADE Rating (RCT) = High. [Critical flaws: Nil].
Azuma et al^59^ (2012; Japan)	Before treatment, the patients showed 38%, 33%, and 20% eye opening values (for 3 types of movement). Two weeks after treatment these figures were 74%, 84%, and 74%. After 10 months the patient showed 80%, 69%, and 67% of % eye opening values.	Single dose of botulinum A toxin followed by facial rehabilitation shows improvement in eye opening
Barth et al^54^ (2020; USA)	Patients in the mirror book therapy group showed an average of 24.9% increase in the Facial Grading System score, a 21.6% increase in the Facial Disability Index–Physical score, and a 24.5% increase in the Facial Disability Index–Social score. Addition of mirror book therapy shows average 20.8% increase in FGS, 19% increase in FDIP, and 14.6% increase in FDIS score.	The addition of mirror book therapy to standard facial rehabilitation treatments significantly improves outcomes in the treatment of idiopathic facial palsy.
Dalla Toffola et al^51^ (2012; Italy)	There was no difference in recovery time, number of treatment sessions or House scores at baseline and 12 months for electromyographic biofeedback and mirror visual feedback groups. The number of days the motor deficit remained unchanged before motor recovery was similar. No significant difference in terms of presence of synkinesis (*P* = 0.63) or severity (*P* = 0.9).	Both groups achieved a similar level of recovery. Patients with neurapraxia recovered without treatment. Clinical outcomes for patients treated with electromyographic biofeedback and mirror visual feedback did not differ.
Dalla Toffola et al^52^ (2014; Italy)	29 patients showed significant decrease in House-Brackmann grade from a value of VI for all patients before surgery, to a median of V (25th-75th V-VI) at the first rehabilitation assessment, and V (25th-75th IV-V), III (25th-75th III-IV) and III (25th-75th III-III) at 12, 18 and 36 months respectively (Friedman test *P* < 0.001).	Patients undergoing long-term rehabilitation programme show significant recovery of facial symmetry and movement, continuing for three years after anastomosis (surgery).
Di Stadio et al^66^ (2020; Italy)	Group A (Kabat rehabilitation), and group B patients (combining facial taping and Kabat) both showed statistically significant within group improvements in ADS scores from baseline, 1 week, 1 month and 3 months after treatment (*P* < 0.0001). Between group analysis shows addition of taping may improve speed of recovery with faster improvement on ADS scale at the 1 month time point (*P* < 0.01), but outcomes at 3 months were identical.	Both groups showed significant improvement from baseline. Addition of facial taping may reduce the time for recovery, but does not lead to overall improved recovery at 6 months. GRADE Rating (RCT) = Low . [Critical flaws: Domain 1 and 2]
Ferreira et al^61^ (2016;Portugal)	Recovery degree and facial symmetry improved significantly in both groups (*P* < 0.001), without differences between groups (*P* > 0.05). Corticosteroid plus facial neuromuscular training (FNT) better outcomes for cheek (*P* < 0.004) and mouth (*P* < 0.022) resting symmetry at SB-FGS, if compared to FNT alone. No significant effect on all recovery degrees (*P* < 0.992) and rapid remission (*P* < 0.952) between the two groups.	No difference in overall recovery and facial symmetry between corticosteroids followed by facial neuromuscular training (FNT) and FNT alone. GRADE Rating (RCT) = High. [Critical flaws: Nil].
Fujiwara et al^53^; (2018; Japan).	All patients had physical therapy and mirror biofeedback and all showed improvement. Both voluntary movement scores and synkinesis scores on the Sunnybrook facial grading system showed significant increases between the 6th and 12th month (*P* < 0.05). Asymmetry in eye opening width showed a slight improvement between the 9^th^ and 12^th^ months. Female patients and younger patients did not show any deterioration in synkinesis. Patients in the lower electroneurography group and the later onset of synkinesis group showed significant deterioration after the 6th month.	Physical rehabilitation shown to prevent significant deterioration in synkinesis in female and younger patients with facial nerve palsy.
Karp et al^55^ (2019; USA)	All 76 patients, presenting 1 to 32 years after onset, showed improvement in FGS scores with facial rehabilitation. Multiple regression model identified significant improvement in FGS score of 1.85 points for each additional therapy session (*P* < 0.001), 0.1 less change in FGS score (*P* = 0.033) for each additional point in the starting FGS score, and right-sided facial paralysis predicted a 4.4-point improvement over left-sided facial nerve paralysis (*P* = 0.008).	Facial rehabilitation was associated with improved FGS score regardless of patient age, gender, or latency (time) to start of facial therapy.
Kim et al^56^ (2011; South Korea)	Symmetric self-performed facial muscle exercises (SSFE) and standard Western therapy showed significant improvement on House–Brackmann Scale and Yanagihara's system (*P* < 0.01). The change of scores in the SSFEs group between baseline and after 4 weeks was greater than that in the control group (HBS *P* < 0.05 and Y *P* < 0.01)	Addition of Symmetric self-performed facial muscle exercises resulted in greater improvement at 4 weeks.
Martineau et al^49^ (2020; Canada)	No significant effect of Mirror Effect PLUS Protocol (MEPP) treatment on Sunnybrook Facial Grading System, and Facial Disability Index outcomes for the entire study sample based on difference of means. At individual level more patients showed progress in SB scale, Speech and swallowing score of FDI at 1 month and 2 month for treatment than control group.	No significant effect of Mirror Effect PLUS Protocol treatment.
Monini et al^64^ (2011; Italy)	Preventive Botulinum toxin type A (BTX-A) treatment group showed 2.1 improvement on SunnyBrook scale (*P* < 0.001), in comparison to the rehab-only group. BTX-A group score for improvement of synkinesis, as defined by the 90 day vs 7 day difference, was 5.4 (95% CI = 4.38–6.42; *P* < 0.001), and partial improvement was 3.3 (*P* < 0.001) versus rehab only group, 3.8 (95% CI = 1.48–4.86; *P* < 0.001).	Addition of Botulinum toxin type A (BTX-A) to standard facial therapy results in greater improvement on the Sunnybrook scale. GRADE Rating (RCT) = Medium. [Critical flaws: Domain 1, 2 and 4]
Monini et al^58^ (2017; Italy)	Corticosteroids only patients, mean duration of clinical recovery >65 years sub-group was 145.1 days; ≤65 years sub-group 103.2 days. In steroid therapy plus Kabat rehabilitation, mean duration of recovery in >65 years sub-group 85 days; ≤65 years sub-group 54.9 days. Beta FaCE Scale total score, pre- vs post-rehabilitation, was not significant in >65 years group; but significant in the ≤65 years sub-group (*P* = 0.008).	Improved outcomes with addition of Kabat rehabilitation to steroid therapy. Recovery especially influenced in >65 years with a severe House–Brackmann grade (HBS Grade V).
Nicastri et al^62^ (2013; Italy)	Significant difference patients reaching House-Brackmann Grade II at 6-months only in severe facial palsy group (HBS Grade V/VI) - control group 11/23 (48%), treatment group 17/23 (74%), *P* = 0.038; and significant difference in time to recovery (*P* = 0.044). No significant differences in synkinesis at 6-months. For patients presenting with HBS grade V-VI, difference in final SunnyBrook scores (*P* = 0.021 Mann-Whitney *U* test).	The physical therapy had a significant effect on HBS grade and time to recovery only in patients presenting with severe facial palsy. GRADE Rating (RCT) = High. [Critical flaw: Domain 1]
Ordahan & Karahan^65^ (2017; Turkey).	Physical exercise only group, FDI scores not significantly improved at week 3 (*P* < 0.05) but improvement at week 6 (*P* < 0.001). In low-level laser therapy and physical therapy group, Facial Disability Index (FDI) scores significantly improved at weeks 3 and 6 (*P* < 0.001). Improvement in laser group significantly greater than those in the exercise group (*P* < 0.05).	Combined treatment with low-level laser therapy and physical exercise therapy associated with significant improvements in FDI when compared with physical exercise therapy alone. GRADE Rating (RCT) = Medium. [Critical flaws: Domain 1, 2, 3 and 4]
Pourmomeny et al^67^ (2015; Iran)	The mean Facial Grading System values using Photoshop software for the Botox group before and after treatment were 55.17 and 74.17, respectively, and those for the biofeedback group were 65.47 and 81.37, respectively. In both groups oral-ocular and oculo-oral synkinesis decreased significantly after treatment compared with before treatment (*P* < 0.01).	Biofeedback rehabilitation therapy and combination biofeedback and Botox appear to have same effect in reducing synkinesis and recovery of face symmetry in Belĺs palsy. GRADE Rating (RCT) = Low. [Critical flaws: Domain 1 and 4]
Rodriguez et al^50^ (2012; Canada)	By four weeks, patients who performed eye exercises improved the strength of their paretic orbicularis oculi muscle more than those who did not (*P* = 0.029). Overall, 63.8% of patients performing exercises (7/11) gauged to have achieved functional recovery at four weeks compared to 12.5% (1/8) of those who did not (*P* = 0.059).	Patients performing exercises achieved greater functional recovery at four weeks compared to those who did not (*P* = 0.059) Eye exercises have a potential role in the treatment of idiopathic cranial nerve VII paresis.
Tuncay et al^63^ (2015; Turkey)	HBS scores at 3 months significantly better for combined electric stimulation plus physical therapy group versus exercise only group (*P* = 0.03). FDI posttreatment scores in combined treatment group statistically higher (physical function, *P* = 0.02; social/well-being function, *P* = 0.03) Mean motor nerve latencies/compound muscle action potential amplitudes of both facial muscles were statistically shorter in electric stimulation group.	Addition of 3 wks. of daily electrical stimulation treatment to physical therapy shortly after facial palsy onset (4 wks.), improved functional facial movements and electrophysiologic outcome measures at 3-month follow-up. GRADE Rating RCT = High. . [Critical flaws: Nil.]

HBS: House–Brackmann Grading System; SB-FGS: SunnyBrook Grading Scale; FDI: Facial Disability Index; GRADE (RCT): 5 Critical Domains. [Domain 1: *Risk of Bias*; Domain 2: *Inconsistency*; Domain 3: *Indirectness*; Domain 4: *Imprecision*; Domain 5: *Publication Bias (other considerations)*].

### Effectiveness of physical rehabilitation

Table 3 summarises the main findings and the conclusions drawn from the 19 new research studies identified. Of the 11 articles assessed to be of medium to high quality, all reported improvements following the use of physical rehabilitation for those with facial palsy.

Among the five high-quality articles, four were randomised controlled studies all of which reported a positive effect. Tuncay et al.^63^ randomised patients with HBS scores of 2–4 into electrical stimulation with exercise or exercise-only treatment groups. They reported a statistically significant improvement in HBS scores at three months after onset in the combined treatment group but no difference in FDI scores between the groups. The exclusion of HBS grades 5 and 6 and the limited sensitivity of the HBS scale makes it difficult to draw any conclusions for these patients. Alayat et al.^60^ found that high-intensity laser therapy, massage, and exercise three times a week for six weeks provided statistically significant improvements in HBS and FDI scores in those in the acute phase of unilateral Bell's Palsy when compared with low-intensity light therapy with massage and exercise or massage and exercise alone; the exercise-only group showed the lowest effect. Nicastri et al.^62^ reported physical therapy produced a significant improvement in HBS grade, but time to recovery only improved in those with severe (HBS 5/6) facial palsy. Ferreira et al.^61^ reported, with the exception of resting symmetry of the cheek and mouth on the SFGS, that neuromuscular retraining was as effective as acute provision of corticosteroids in improving static and dynamic symmetry in those with unilateral idiopathic facial palsy. The final, observational study, Dalla Toffola et al.^51^ found no difference in the recovery in patients with idiopathic facial palsy and evidence of axonometric facial nerve injury when provided with EMG biofeedback compared to mirror biofeedback as a primary rehabilitation tool. Development of synkinesis, extent of motor recovery, and health resources used was included in their appraisal of recovery.

The six articles rated as medium quality included two randomised controlled trials and four non-experimental observational studies. All reported improvements Monini et al.^64^ completed a small trial of 20 consecutive patients randomly allocated to pre-treatment with Botulinum toxin type-A prior to standard facial therapy. They found that both groups showed improved outcomes on the SunnyBrook scale, but pre-treated patients had significantly better outcomes. Ordahan and Karahan^65^ randomized 46 patients with acute idiopathic palsy to physical exercise with or without low-level laser therapy. They reported that therapy alone showed a significant improvement in FDI score from baseline at week 6 but not at week 3. Laser therapy and exercise produced significant improvements from baseline FDI scores at both three weeks and six weeks post onset.

Among the four observational studies rated as medium quality, Barth et al.^54^ reported a statistically significant increase in SFGS, FDIP and FDIS when mirror therapy was combined with standard facial rehabilitation. Standard rehabilitation was documented as including massage, stretching, neuromuscular re-education, myofascial release, and postural exercises. Monini et al.^58^ found that treating all age groups with steroid therapy and physical rehabilitation resulted in a significantly quicker time to recovery than steroid therapy alone. They also highlighted the importance of physical therapy for older patients and those with more severe HBS grading at the onset of their palsy. This followed their previous trial showing standard facial therapy results were improved with the addition of preventive Botulinum toxin treatment.^64^ Kim et al.^56^ compared Western physical therapy with Korean symmetric self-performed facial muscle exercises, reporting that the addition of SSFE showed significant improvement in HBS at four weeks.^56^ In a study by Fujiwara et al.,^53^ physical rehabilitation was shown to prevent significant deterioration in synkinesis in female and younger patients with Bell's palsy.

Eight of the 11 individual studies assessed to be of medium to high quality included the statistical significance of their results (*P* values). One reported a non-significant difference between two methods of biofeedback (electromyographic and mirror visual feedback),^51^ while all others demonstrated a significant improvement.^53,56,58,61–65^ No studies attempted to calculate effect size, so there were insufficient data to assess imprecision or inconsistency as outlined in GRADE approach.^39^

Among the remaining nine individual research studies that were assessed to be of low quality, three articles reported a statistically significant effect ranging from *P* < 0.05 to *P* < 0.001,^52,55,57^ Barth et al.^54^ reported changes were ‘statistically significant’, one study (a small pilot trial) reported no significant effect,^49^ and two studies simply reported percentage improvements.^48,59^ In the one poor quality randomised controlled trial, Di Stadio et al.^66^ reported a significant improvement for Kabat rehabilitation with and without facial taping based on a new rating Arianna Disease Scale.^69^

### Timescale and sustainability of therapeutic effects

The timescales over which physical therapy was assessed differed widely and this limits any robust analysis of how sustainable the reported therapeutic effects were. Dalla Toffola et al.^52^ compared the efficacy of EMG and mirror biofeedback over a 12-month period. Ferreira et al.^61^ assessed the role of corticosteroids (prednisolone) and neuromuscular retraining in recovery from acute idiopathic palsy and so chose to perform their analyses at the entry to the study and six weeks later. Kim et al.^56^ reviewed acute outcomes at four weeks post onset. Fujiwara et al.^53^ appraising physical therapy in the treatment of synkinesis selected 6, 9 and 12-month intervals to assess the outcomes. Nicastri et al.^62^ followed study participants up on a monthly basis for six months.

### Evidence from systematic reviews

Table 4 presents an overview of the six reviews shortlisted for examination in publication date order, with data from the original Cochrane review included for comparison.^32^ The six new reviews were published over the period 2011–2017 and originated from research groups in Australia,^42^ Brazil,^43^ Iran,^44^ Italy,^45^ New Zealand,^46^ and Portugal.^47^ Apart from the 2011 Cochrane review by Teixeira et al., all systematic reviews were rated as ‘critically low’ using the AMSTAR-2 rating.^40^

Table 5 summarises the main findings and conclusions drawn by the authors, and the weaknesses identified in these reviews. The one high-quality review remains that was undertaken by Teixeira et al.^32^ This found low-quality evidence that tailored facial exercise could reduce sequelae in the acute phase, could improve facial function in those with moderate paralysis, and could produce improvements in the chronic stages. The authors concluded that further research evidence was required, as provided by individual studies listed in Tables 2 and 3, none of which are included in the Teixeira review.

**Table 5.** Findings reported by included reviews.

Review Authors (date/country)	Review Main Findings	Review Conclusions/AMSTAR-2 Rating
Teixeira et al^32^ (2011; Brazil)	Electrostimulation produced no benefit over placebo on the primary outcome of incomplete recovery (moderate quality evidence from one study with 86 participants). Low quality comparisons of electrostimulation with prednisolone (an active treatment) (149 participants), or the addition of electrostimulation to hot packs, massage and facial exercises (22 participants), reported no significant differences on incomplete recovery. There was moderate quality evidence for positive effects of facial exercises on secondary outcomes of facial disability (34 participants) and low quality evidence for positive effects on motor synkinesis in acute cases (145 participants). There was low quality evidence for positive effects on time for complete recovery in more severe cases (47 participants).	AMSTAR-2 Rating = High. [Critical flaws: None] Concludes: Evidence that tailored facial exercises can improve facial function (low quality), mainly for people with moderate paralysis and chronic cases; and can reduce sequelae in acute cases.
Pereira et al^43^ (2011; Brazil).	Mean and standard deviation showing improved functionality for facial exercise therapy (pre- and post-treatment) reported in three randomized controlled trials. One study had sufficient data for meta-analysis; improvements in facial grading scale at 3 months in facial exercise therapy group (standardized mean difference = 13.90; 95% confidence interval (CI) 4.31, 23.49; *P* = 0.005	AMSTAR-2 Rating = Critically low. [Critical flaws: Domains 2, 7, 11, 13, 15 (plus only ‘partial yes’ to items 4, 9)] Concludes: Facial exercise therapy is effective for facial palsy for the outcome functionality.
Baricich et al.^45^ (2012; Italy) Have full article now	Mime therapy: One study of moderate risk of bias (American Academy of Neurology Class II) found significant improvements in HBS and SB-FGS after 3 months of treatment relative to a control group. Other interventions: Four studies with very high risk of bias (Class IV) found evidence for electrostimulation, biofeedback, proprioceptive neuromuscular facilitation, and neuromuscular re-education either over time or relative to a control group.	AMSTAR-2 Rating = Critically low. [Critical flaws: Domains 2, 4, 7, 9] Concludes: Moderate level evidence for the effectiveness of mime therapy in peripheral facial paralysis.
Holland et al.^46^ (2014; New Zealand)	Weak evidence for facial re-training using mime therapy or exercise versus a waiting list control. Mime Therapy (RCT evidence): FDI score (*P* < 0.02); FDI social score (*P* < 0.01); stiffness score (*P* < 0.001); pout score (*P* < 0.001); lip length score (*P* < 0.03). Exercises vs waiting list (systematic review evidence): SB-FGS average difference 20.40; 95% CI [8.76, 32.04]; FDI social domain average difference 14.50, 95% CI [4.85, 24.15]	AMSTAR-2 Rating = Critically low. [Critical flaws: Domains 4, 7, 9 (plus only ‘partial yes’ to item 2)] Concludes: Facial re-training using mime therapy or exercise may be more effective than waiting list control at improving facial function scores at 3 months, but no more effective at reducing risk of incomplete recovery at 3 months.
Pourmomeny & Asadi^44^ (2014; Iran).	All of the studies reviewed demonstrate that facial exercise therapy is effective in terms of facial symmetry. Four studies reported EMG biofeedback is effective through neuromuscular re-education.	AMSTAR-2 Rating = Critically low. [Critical flaws: Domains 2, 4, 7, 9, 13] Concludes: For patients with incomplete recovery of facial nerve paralysis, facial exercise therapy is effective, with most evidence for the value of electromyogram (EMG) biofeedback.
Ferreira et al.^47^ (2015; Portugal)	Three trials (one rated ‘good’ and two ‘fair’ methodological quality) indicated that physical therapy (PT) in association with standard drug treatment supports higher motor function recovery than standard drug treatment alone between 15 days and 1 year of follow-up. One trial (rated ‘poor’ methodological quality) showed that electrical stimulation added to conventional PT with SDT did not influence treatment outcomes.	AMSTAR-2 Rating = Critically low. [Critical flaws: Domains 2, 13 (plus only ‘partial yes’ to items 4, 9)]. Concludes: Bell's palsy treatment by physical therapy and standard drug treatment seems to have a positive effect on grade and time to recovery compared with drug treatment alone. However, very little quality RCT evidence, so insufficient to decide whether combined treatment is beneficial.
Fargher & Coulson^42^ (2017; Australia)	In the acute phase, electrical stimulation does not alter the speed or rate of full recovery, or improve facial function. Meta-analysis on changes in facial function showed no statistically significant difference between intervention and control groups (HBS). In the chronic phase of recovery, there is low quality evidence in one study that extensive electrical stimulation may have a positive impact on facial function (Facial Paralysis Recovery Profile).	AMSTAR-2 Rating = Critically low. [Critical flaws: Domains 2, 13, 15] Concludes: No scientific evidence to support electrostimulation during recovery from the acute phase of Bell's palsy. One study provides low level of evidence in patients with chronic symptoms.

CMAPs: Compound Muscle Action Potentials; EMG: electromyography; FDI: Facial Disability Index; HBS: House–Brackmann Scale; SB-FGS: Sunnybrook Facial Grading System; AMSTAR-2: 15 Critical Domains [Domain 2: *Explicit protocol prior to the conduct of review*; Domain 4: *Comprehensive literature search strategy*; Domain 7: *List of excluded studies with justification*; Domain 9: *Satisfactory technique for assessing risk of bias*; Domain 11: *If meta-analysis performed, appropriate methods for statistical combination*; Domain 13: *Account for risk of bias when interpreting/discussing results of individual studies*; Domain 15: *If quantitative synthesis performed, investigation of publication bias (small study bias)*.

The six more recent reviews all reach largely positive conclusions about the effectiveness of facial exercise therapy.^42–47^ But all have major methodological shortcomings resulting in critically low AMSTAR-2 quality ratings, so could not be included in our synthesis. Critical weaknesses include lack of clarity *a priori* about the review protocol; deviations from the original protocol; a lack of comprehensive description of search strategies, excluded studies, or risk of bias assessments; and no consideration of how bias might influence the interpretation of results. Equally important, the reviews did not include additional new evidence; only half identified any studies published since the 2011 Teixeira review. Pourmomeny and Asadi^44^ identified two new randomised controlled trials; Ferreira et al.^47^ included one new study; and the 2017 review by Fargher and Coulson^42^ identified two randomised controlled trials. Only three of these five studies met our inclusion criteria and they had already been included in our review^51,62,63^; the other two did not focus on facial exercise therapy.^70,71^

## Discussion

The primary aim of this study was to update the evidence base on the effectiveness of physical therapy for facial palsy rehabilitation,^32^ focusing on tailored facial exercise therapy. The review identified nineteen new research studies, including eight randomised controlled trials, four quasi-experimental or pilot studies, and seven observational studies. These studies provide evidence on the effectiveness of facial exercise therapy in 839 patients with ages ranging from 12 to 80 years old. Although, six reviews published after the Cochrane review were also identified all were rated as being of critically low quality. They also identified only three new studies which met our inclusion criteria, and all these were already included in our review. This article therefore adds significantly to previously available evidence on the effectiveness of facial exercise therapy for the recovery of people diagnosed with facial palsy.

The 19 new studies included in this review vary in their research design. Seven incorporated a randomised controlled trial, nine used a non-experimental observational design, and the remainder were quasi-experimental or pilot studies. Overall, four out of seven trials were rated as high quality and all described a positive impact. Among the nine observational studies, three were rated as high or moderate quality, all cohort studies. Two of these studies reported a significantly better outcome for those receiving facial exercise therapy, especially among younger patients.^53,58^

The effectiveness of facial exercise therapy on its own was evaluated in only two studies; both reported that patients performing facial exercises achieved greater functional recovery than those who did not.^50,55^ Six studies assessed physical therapy combined with biofeedback, either via a mirror or other means; all reported positive improvements.^49,51–54,66^

The remaining 10 studies evaluated the effectiveness of combining facial exercise therapy with corticosteroids,^58,61,62^ botulinum toxin,^57,59,64^ electrical stimulation,^48,63^ or laser treatment.^60,65^ Nine reported a positive improvement.

This review considerably strengthens the evidence base in support of facial exercise therapy, but information on the specific benefits of therapy at different timepoints post-onset of facial palsy or for patients living with different levels of severity is difficult to disentangle with certainty. In studies where the stage of recovery was specified, six studies concentrated on the acute phase, recruiting patients from 48 h to one month since onset of facial paralysis.^48,51,60,62,63,66^ A further 12 studies focused on chronic cases including patients from 2 to 30 years post onset.^55,64^ These studies strengthen previous Cochrane review evidence on the value of physical therapy early in recovery, and also add to the previously limited evidence in support of its use for chronic cases.^32^ Evidence of the specific benefits with increased clinical severity is less clear. Five research studies selected patients based on the severity of their facial palsy, with cut-offs ranging from Grade II to VII,^50,52,58,62,64^ and a further three reported selecting patients based on the presence of facial synkinesis.^53,57,59^ However, it is not possible to draw any conclusions on the impact of physical therapy depending on the clinical severity of facial palsy from these studies.

The review findings also demonstrate an increase in the use of validated outcome measures, compared to research identified in the earlier Cochrane review.^32^ A clinician facial grading system (House–Brackmann or Sunnybrook) was used in 12 studies, and a patient-rated outcome measure (Facial Disability Index) was included in six studies. The use of both a clinician and patient outcome measure was observed in the highly rated studies. A recent systematic review has recommended the adoption of Sunnybrook for reporting outcomes of facial nerve disorders.^72^ Only three studies included in the review used a single, non-validated outcome measure; all were rated low quality. No study defined *ex ante* what would represent a clinically significant change. A similar finding has been reported in the service setting when monitoring patient recovery.^20^ No study included cost-effectiveness as an outcome or incorporated a health-utility outcome measure (e.g. EQ-5D) that would enable economic modelling.^73^ Finally, because the point at which outcomes were measured ranged from 4 weeks to 12 months post-treatment, evidence on the sustainability of any improvements following therapy is difficult to gauge. Arguably, a comprehensive analysis of the impact of therapy on the evolution of facial palsy, including synkinesis, should be performed over a minimum of a 24-month period.^74^

## Conclusions and future influence on clinical practice

This review provides new evidence from high-quality studies to support the use of facial exercise therapy in patients with facial palsy. Limitations of the evidence include differences in research design, the interventions evaluated, and patients included in studies. This heterogeneity makes it impossible to pool data from individual studies to increase the power and precision of estimates of treatment effects.^68^ The heterogeneous nature of the current evidence base also limits the ability to draw any clear correlation between the effectiveness of facial exercise therapy and time since onset of facial palsy, clinical severity, or other patient demographics. The use of both a clinician grading system and patient-rated outcome measure, as identified in high-quality studies, is recommended.

Looking to the future, further improvements to the evidence-base will become even more important if reports emerging of a link to COVID-19 are substantiated. These include a tripling of cases in patients who have had COVID-19,^75^ as well as possible links to vaccines,^76,77^ with safety concerns already officially recorded for the latter,^78^ and a recent call for surveillance following use of particular vaccines.^79^ Large-scale COVID-19 vaccine programmes could lead to a rise internationally in the future number of facial palsy cases. Our review demonstrates widespread international interest in evaluation of physical therapy for facial palsy, with studies originating from 10 different countries. This provides a good foundation for future collaborative research, moving away from a previous pattern of studies almost exclusively undertaken in the United States.^31^ International collaborations are already leading to standardisation of outcome measures, and development of value-based reimbursement strategies.^80^ A future move to digitally enabled facial palsy rehabilitation at home is also likely to be catalysed.^20,34^ Pre-pandemic, a review of telerehabilitation could identify no facial palsy studies.^81^ This situation is likely to change as a consequence of an accelerated worldwide move to digitally enable services due to COVID-19,^82^ new routes for commissioning home-based digital services,^83,84^ and the development of new wearable technologies.^33^

This review has concentrated on peripheral facial nerve paralysis. A similar evidence base for facial palsy following stroke is currently lacking.^85^ Because this form of central facial palsy is clinically distinct,^86^ current review findings are not directly transferrable. But, the lessons learned here might be applied to improve the evidence base for these patients.

**Clinical messages**

- RCTs of facial exercise therapy confirm improved facial functioning and patient-reported outcomes.
- Therapy is effective in early cases, when combined with other treatments, and potentially in chronic cases.
- Multi-centre collaboration is needed, particularly given emerging evidence of links with COVID vaccination.
- Specialist facial therapy should be integrated fully into treatment pathways.
